# SAR and Optical Data Comparison for Detecting Co-Seismic Slip and Induced Phenomena during the 2018 M_w_ 7.5 Sulawesi Earthquake

**DOI:** 10.3390/s19183976

**Published:** 2019-09-14

**Authors:** Marco Polcari, Cristiano Tolomei, Christian Bignami, Salvatore Stramondo

**Affiliations:** Istituto Nazionale di Geofisica e Vulcanologia, Via di Vigna Murata 605, 00143 Rome, Italy; cristiano.tolomei@ingv.it (C.T.); christian.bignami@ingv.it (C.B.); salvatore.stramondo@ingv.it (S.S.)

**Keywords:** Sulawesi earthquake, Palu-Koro fault, SAR data, optical data, SAR Interferometry, ixel Offset Tracking, Sub-pixel correlation

## Abstract

We use both Synthetic Aperture Radar (SAR) and Optical data to constrain the co-seismic ground deformation produced by the 2018 M_w_ 7.5 Sulawesi earthquake. We exploit data processing techniques mainly based on pixel cross-correlation approach, applied to Synthetic Aperture Radar (SAR) and optical images to estimate the North–South (NS) displacement component. This component is the most significant because of the NNW–SSE geometry of the fault responsible for the seismic event, i.e., the Palu-Koro fault, characterized by a strike-slip faulting mechanism. Our results show a good agreement between the different data allowing to clearly identify the surface rupture due to the fault slip. Moreover, we use SAR and optical intensity images to investigate several secondary phenomena generated by the seismic event such as tsunami, landslides, and coastal retreat. Finally, we discuss differences between SAR and optical outcomes showing strengths and disadvantages of each one according to the investigated phenomenon.

## 1. Introduction

On 28 September 2018, a large M_w_ 7.5 earthquake struck the Sulawesi island, Indonesia, producing big tsunamis with waves higher than 2 m, landslides, flooding, and soil liquefaction effects which severely affected Palu Bay [1,2,3,4]. The estimated kinematics indicates how the seismic event nucleated along a fault showing a strike-slip mechanism at a depth of 15–20 km with the epicenter located about 80 km north of Palu city [5]. Most of damages were observed in the urban area of Palu and in the nearby villages where the seismic event and the following induced phenomena destroyed hundreds of buildings causing more than 2000 casualties and 4000 injuries.

The tectonic setting of the area is very complex being Indonesia the largest island country in the world with about 2 million square kilometers split into more than seventeen thousand islands born along the boundaries of several tectonic plates. In particular, Sulawesi is located west of the Maluku Islands and east of Borneo, along a junction between three major plates, i.e., the Australian, Philippine, and Sunda plates. It is one of the four Greater Sunda Islands within the Malay Archipelago, apart from Java, Sumatra, and Borneo islands. Its landmass is formed by four peninsulas, which are the Minahasa Peninsula, the East Peninsula, the South Peninsula, and the South-East Peninsula and divided into six districts, i.e., North Sulawesi, Gorontalo, Central Sulawesi, West Sulawesi, South Sulawesi, and South-East Sulawesi. 

In the proximity of the island, the northward moving Australian plate and the westward moving Philippine plate subduct under the Sunda plate with rates of the order of 5–10 cm/year [6,7]. Such relative motion between plates generates crustal block rotations and transpressive faulting zones with several active structures crossing Sulawesi Island. One of those, the left-lateral NNW–SSE trending Palu-Koro strike-slip fault, cuts Palu Bay through the northeastern part of the island and is responsible for the M_w_ 7.5 seismic event occurred in the western part of Central Sulawesi.

**Figure 1:**
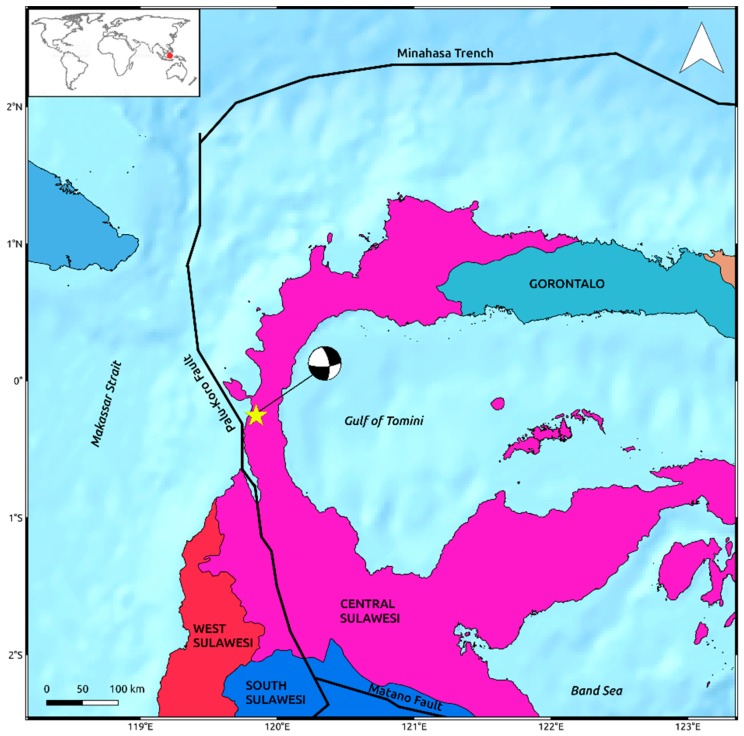
Overview on landmass and tectonic settings of Sulawesi island. Solid lines are the main tectonic structures crossing the island retrieved by the Global Earthquake Model (GEM) Global Active Faults project [16]. The yellow star indicates the epicenter of the M_w_ 7.5 2018 Sulawesi earthquake occurred on the western part of Central Sulawesi district. The focal mechanism is retrieved by the United States Geological Survey (USGS) [5].

The Palu-Koro fault separates the two major microblocks forming Sulawesi Island, i.e., the Makassar and North Sula blocks, and accommodates about 40 mm/year of relative crustal blocks motion [7]. To the south, it connects with another important structure of the island, the WNW–ESE Matano fault, whereas it extends northward crossing Palu Bay and passing to the west of the Minahasa Peninsula before connecting with the Minahasa Trench along North Sulawesi Subduction Zone (Figure 1). Such fault is already known to be highly active and the associated seismicity has been largely monitored in several studies [8,9,10].

During the M_w_ 7.5 seismic event it ruptured, with evidences of supershear, starting along a complex and a previously unmapped segment to the north, just south the epicenter, and then following along its almost linear mapped segment from Palu Bay to the south [11,12].

The aim of this work is to evaluate the performance of satellite remote sensing data in estimating the co-seismic ground deformation and secondary phenomena produced by the slip of Palu-Koro fault. To this purpose, we applied Synthetic Aperture Radar (SAR) Interferometry (InSAR) [13] and Pixel Offset Tracking (POT) [14] techniques to a pair of both Sentinel-1 and ALOS-2 Synthetic Aperture Radar (SAR) acquisitions and Sub-Pixel correlation (SBP) [15] method to a couple of Sentinel-2 optical images constraining the seismic event. In addition, we also exploited the pre- and post-event intensity images for evaluating the effects of seismic-induced phenomena on SAR and optical scattering signal. 

## 2. SAR Data

The SAR data used in this work consist in a couple of C-band SAR images acquired along descending track on 7 June and 11 October, 2018 from S1 missions of the European Space Agency (ESA) and a couple of L-band Wide-Swath descending SAR images acquired by ALOS-2 mission of the Japan Aerospace Exploration Agency (JAXA) on 21 August and 2 October. The two acquisition geometries are quite similar consisting of an incidence angle of ~34° for Sentinel-1 images and ~29° for ALOS-2 ones and an azimuth angle of about −168° for both of them. 

We firstly applied multi-look factors such as to retrieve the same ground pixel spacing of about 100 m along range and azimuth direction for both Sentinel-1 and ALOS-2 data. This allows to significantly improve the signal-to-noise ratio and also to approximately obtain the size of Digital Elevation Model (DEM) provided by the Shuttle Radar Topography Mission (SRTM) [17] and exploited for removing the topography in InSAR processing and for geocoding step. Instead, when working on the intensity images, we used smaller multi-look factors in order to get squared pixels (about 15 m) but preserving as much as possible any details of the investigated local-scale phenomena.

### 2.1. InSAR Outcomes

We adopted the standard InSAR technique [13] for processing both C-band Sentinel-1 and L-Band ALOS-2 SAR data. Then, we performed images coregistration step, interferogram calculation and filtering by using the software packages developed by GAMMA Remote Sensing and Consulting © [18].

**Figure 2:**
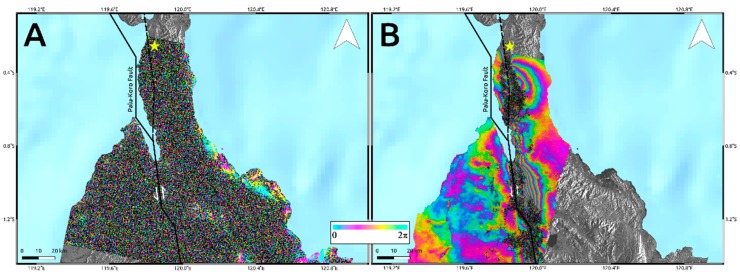
Wrapped interferogram retrieved by Sentinel-1 (**A**) and ALOS-2 (**B**) data. Each color cycle represents a LoS displacement equal to λ/2. The figure highlights the unsuitability of C-band sensors to capture high spatial rates of deformation as in the case of the Sulawesi earthquake. Solid lines are the main tectonic structures crossing the island retrieved by the GEM Global Active Faults project [16]. The dashed line is the unmapped segment of the Palu-Koro fault that ruptured during the seismic event. The yellow star indicates the epicenter of the Mw 7.5 2018 Sulawesi earthquake occurred on the western part of Central Sulawesi district.

**Figure 3:**
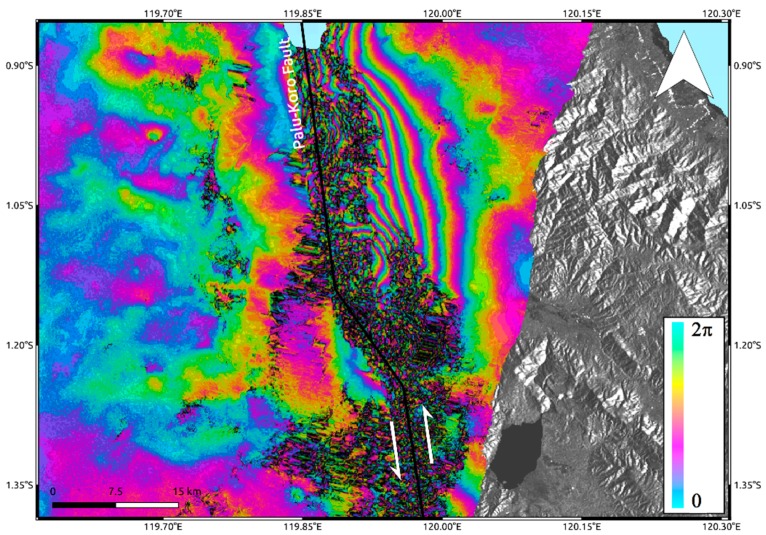
Focus on L-band interferogram in the proximity of Palu-Koro fault rupture trace.

Unfortunately, the wide surface displacement due to the M_w_ 7.5 Sulawesi earthquake produced strong ambiguity problems in InSAR results. This is because InSAR products consist of wrapped phase maps where every phase color cycle (2π), also called interferometric fringe, represents a deformation equal to half wavelength of the sensor working frequency, measured in the Line of Sight (LoS) of the sensor itself. Therefore, if a deformation phenomenon is very impressive, it could not be possible to recognize and discriminate the interferometric fringes thus leading to a signal loss. Indeed, although we applied very strong Goldstein filtering [19], this technique is not able to recover signal especially in the proximity of the fault rupture, i.e., where the deformation reaches its peak. This is particularly evident for Sentinel-1 data (Figure 2A), since half wavelength at C-band corresponds to ~2.83 cm. In addition, C-band is also more sensitive to temporal decorrelation effects induced by large vegetated areas surrounding the urban area of Palu. On the other hand, because of the larger half wavelength, i.e., ~12 cm, L-band ALOS-2 data are less sensitive to both phase ambiguity problems and temporal decorrelation effects. Accordingly, L-band interferogram (Figure 2B) shows some interferometric fringes starting from about 6 km far from the Palu-Koro fault trace representing a LoS deformation of more than 60 cm. However, they also do not allow retrieving any information about the near deformation field as clearly observable in Figure 3. Moreover, the retrieved LoS displacement values largely under-estimate the real deformation since most of the displacement occurred along N–S direction, according to the NNW–SSE strike-slip Palu-Koro faulting mechanism, that is the path to which InSAR is less sensitive.

We think that in this particular case, InSAR data are not completely reliable for providing a clear indication of the surface fracturing and to retrieve information about the causative fault. Rather they can give a qualitative evaluation of the deformation pattern to integrate with other source of data for supporting the study of kinematic and speed of rupture propagation of the M_w_ 7.5 Sulawesi earthquake [12,20,21]. 

### 2.2. POT Outcomes

Unlike InSAR data, the Pixel Offset Tracking (POT) technique [14] can work on the intensity of the SAR images, thus it is not affected by phase problems. This technique measures the 2D registration shifts between corresponding pixels of two SAR images, based on the Normalized Cross Correlation (NCC) maximization within a chosen matching window. In particular, common features characterized by strong intensity value along the two images, such as buildings, roads, bridges, or artificial corner reflectors, are exploited to search the shifts between images such as to maximize the NCC parameter. Such registration shifts can be due to real ground surface movements or induced by some artifacts, such as ionospheric effects or orbital errors. However, in most cases by using precise orbit information and under some assumptions, such artifacts can be neglected. Therefore, the offsets between SAR images, estimated along both LoS (i.e., on range direction) and Azimuth, represent any surface deformations occurred along these two directions in the time interval between acquisitions. The accuracy of the estimates strongly depend on the presence of common features and typically ranges between 1/10–1/100 of the spatial resolution, i.e., an order of magnitude worse than InSAR products [22]. Despite the worse accuracy, POT data can be useful in the study of big earthquakes producing large displacement field influencing InSAR phase signal. Moreover, the azimuth displacement component is poorly constrained by InSAR data. Therefore, in this case POT azimuth displacement is the most suitable product in constraining the co-seismic offset due to the M_w_ 7.5 2018 Sulawesi earthquake, being consistent with the NNW–SSE geometry of the Palu-Koro causative fault. 

**Figure 4:**
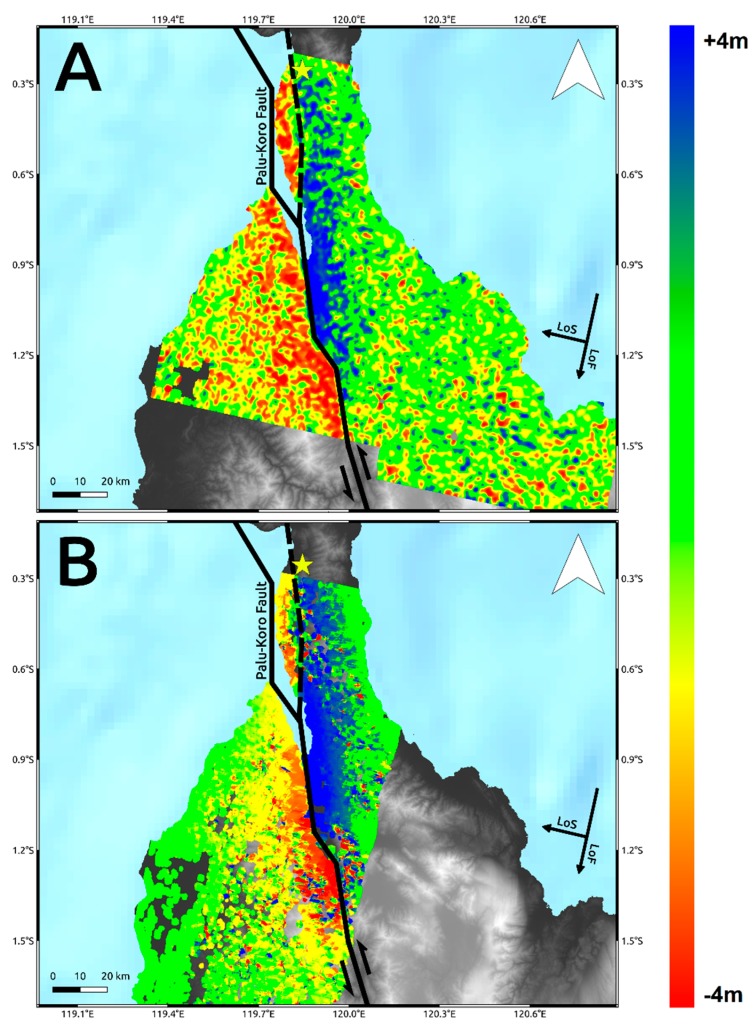
Pixel Offset Tracking (POT) Azimuth displacement retrieved by C-band Sentinel-1 (**A**) and L-band ALOS-2 (**B**) Synthetic Aperture Radar (SAR) data. Solid lines are the main tectonic structures crossing the island retrieved by the GEM Global Active Faults project [16]. The dashed line is the unmapped segment of the Palu-Koro fault that ruptured during the seismic event. The yellow star represents the epicenter of the M_w_ 7.5 earthquake. The background image is the Digital Elevation Model (DEM) provided by the Shuttle Radar Topography Mission (SRTM) [17].

Then, we processed the SAR dataset by POT approach using the working packages implemented in the GAMMA software [18]. An important step in POT analysis is the choice of the matching window size. This is because the pixel spacing of POT products has to be chosen as a fraction of the matching window size, which is typically tens of radar resolution [23]. We performed several tests by varying the matching window size starting from 200 m since we multi-looked all data to have the same pixel spacing of 100 × 100 m. Smaller matching windows returned several solutions affected by errors since it is more sensitive to any local noise. Conversely, bigger matching windows produce a more reliable large-scale solution but local deformation phenomena could be masked by the main and dominant displacement field. Here we are mainly interested in the estimation of co-seismic slip induced by the earthquake then we finally fixed the matching window size to 1500 m for both C-band S-1 and L-band ALOS-2 data, which is about 15 times the pixel spacing. The retrieved POT azimuth displacement is affected by “salt-and-pepper noise” due to several offset estimates carried out on points with low signal-to-noise ratio. Therefore, we refined the result by masking out all pixels with cross-correlation values lower than 0.2 and displacement values out of the range +/−8 m and then by applying a spatial filtering with a windows size of 200 m. The final results for both Sentinel-1 and ALOS-2 data are shown in Figure 4.

## 3. Optical Data

Concerning the optical data, we selected two images acquired on 27 September and 2 October, 2018 from the ESA Sentinel-2 mission. Such images are free and directly available in the ESA Geohazard Exploitation Platform (GEP) of ESA [24], and are acquired with a spatial resolution of about 10m. Also in this case, we considered pixel offsets methodology to investigate the surface deformation field due to the large mainshock. The selected acquisitions pre- and post- earthquake were processed through the tool Mutiple Pairwise Image Correlation of OPtical image Time-series (MPIC-OPT) [25], a service provided in the framework of the ESA GEP. Like all the Pixel Offsets approach, the MPIC-OPT is able to observe and measure horizontal ground movements based on a Sub-pixel image correlation method [15]. Similarly to POT for SAR data, such technique allows obtaining ground offsets with a theoretical accuracy of 1/10 of pixel among homologous pixels by means of correlation methods. Several processing parameters have to be adjusted according to the studied phenomenon. Likewise SAR data, particular care is required for the choice of the matching window size since a smaller window allows to better constrain small scale phenomena but are more sensitive to noise. On the other hand, a larger window lead to greater robustness against noise but small details can be easily smoothed. Here, we fixed the window size to 7 × 7 pixel, i.e., about 70 m, which is a recommended value for most of the applications. Moreover, we discarded all the matches with a correlation coefficient lower than 0.2 by setting the decorrelation threshold accordingly, whereas all the other default parameters are accepted. 

The retrieved shift differences between pixels belonging to the two images return the displacement, already projected along the NS and the EW component, occurred in the time span interval covered by the two considered acquisitions.

**Figure 5:**
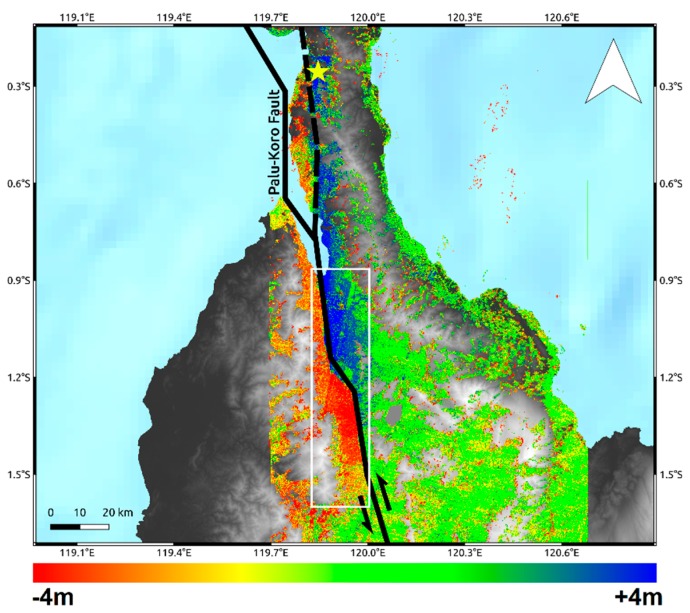
N–S displacement retrieved by C-band Sentinel-2 data. Solid lines are the main tectonic structures crossing the island retrieved by the GEM Global Active Faults project [16]. The dashed line is the unmapped segment of the Palu-Koro fault that ruptured during the seismic event. The yellow star represents the epicenter of the M_w_ 7.5 earthquake. The background image is the DEM provided by SRTM mission [17]. The white rectangle is used to carry out the zonal statistics in Table 1.

According to the NNW–SSE geometry of the Palu-Koro causative fault, the retrieved N–S displacement highlight a quasi-linear trace at the southern part of the Palu urban area and a more complex deformation pattern at the northern part, showing a rupture length of more than 130 km (Figure 5). 

## 4. Results

**Figure 6:**
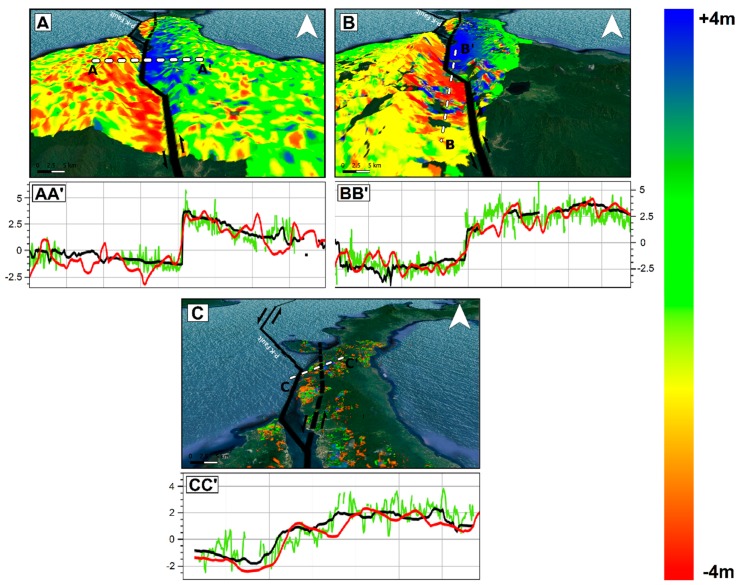
Data profiles showing ground deformations by Sentinel-1 (red), ALOS-2 (black), and Sentinel-2 (green) along AA’, BB’, and CC’ transects. The background maps are the displacement fields retrieved by Sentinel-1 (**A**), ALOS-2 (**B**), and Sentinel-2 (**C**) data.

The results in terms of co-seismic ground displacement retrieved by SAR and optical data are consistent each other both showing a significant co-seismic slip mostly characterized by left-lateral strike-slip component consistently with the Palu-Koro faulting mechanism. Both SAR and optical data show how the causative fault ruptured onshore along at least two segments. The first segment is located in the northern part of Palu Bay just south of the epicenter and extends for about 65 km. It is an unmapped segment of the Palu-Koro fault system and during the seismic event slipped with values ranging from about 1 to 3 m. On the other hand, the second onshore segment starts in Palu bay and extends southward slipping for about 70 km. Remote sensing data show how it clearly corresponds to the mapped trace of Palu-Koro fault, with slip values spanning from about 4–6 m, i.e., at a greater magnitude with respect to the first segment. The trace of the surface fracturing of Palu-Koro fault is well identified by the deformation gradient clearly observable in Sentinel-1, ALOS-2, and Sentinel-2 maps. This is also shown in Figure 6 where we show three data profiles, AA’, BB’, and CC’ to highlight the behavior of SAR and optical data crossing the fault rupture. The remarkable transition between positive and negative displacement is clearly visible and well constrained along all the three transects and also the position and the magnitude of the rupture are in agreement among the different data. Moreover, we estimated the statistics of the retrieved deformation patterns within a polygon of about 70 × 15 km, along N–S and E–W direction, surrounding the Palu-Koro trace fault (white rectangle in Figure 5). We found consistent values and the results of such analysis are summarized in Table 1. In particular, we observed ~6 m of maximum co-seismic offset some kilometers south to the urban area of Palu consistently with analysis carried out by PVMBG-CVGHM (Pusat Vulkanologi dan Mitigasi Bencana Geologi) showing a sinistral offset ranging from 4.6 to 5.8 m [26].

**Figure 7:**
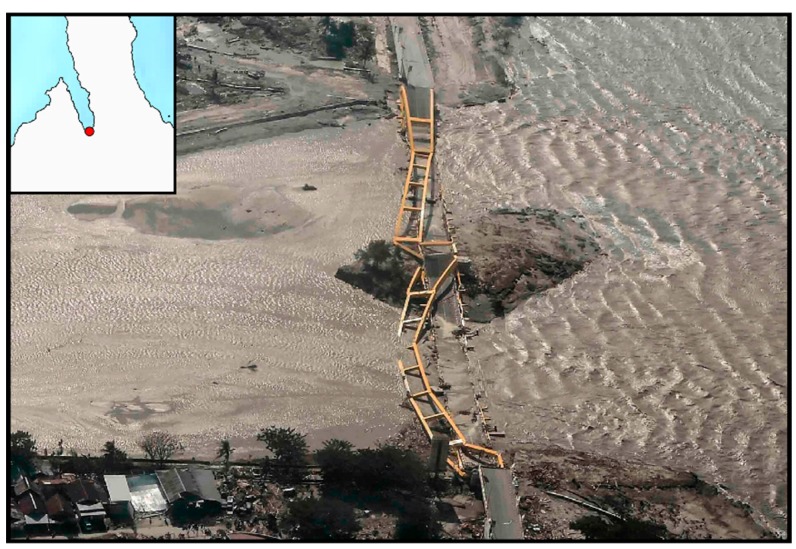
The Palu IV bridge destroyed by the M_w_ 7.5 seismic event and the following tsunami (modified from https://www.nytimes.com/2018/09/30/world/asia/indonesia-tsunami-science.html).

As largely known, such a wide deformation field produced a range of seismic-induced phenomena such as tsunami, soil liquefactions, and landslides correlated to the M_w_ 7.5 Sulawesi earthquake [1,2,3,4]. In particular, after the seismic event, two big landslides have been observed close to the airport and on the eastern outskirts of Palu city [27,28]. Moreover, the M_w_ 7.5 seismic event and the induced phenomena largely impacted on Palu bay provoking a coastline retreat and completely destroying several buildings and the Palu IV bridge [29,30,31] (Figure 7). 

**Figure 8:**
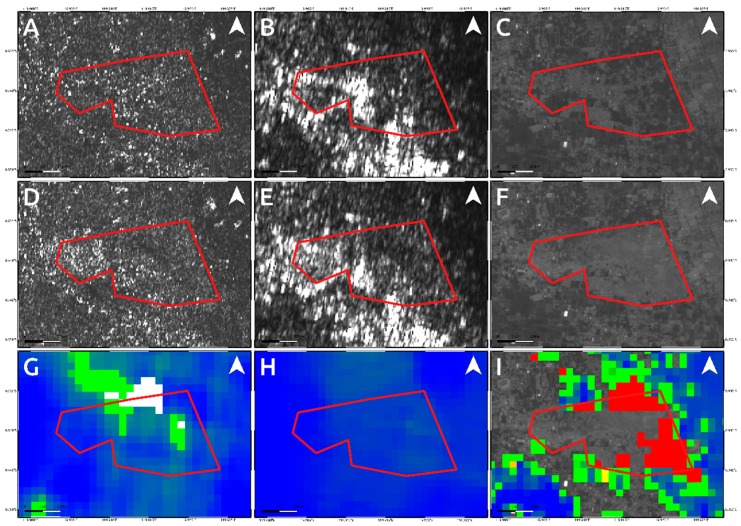
Data comparison for detecting the landslides activated by the M_w_ 7.5 2018 Sulawesi earthquake close to the Palu airport. Upper panels show the pre-seismic image for Sentinel-1 (**A**), ALOS-2 (**B**) and Sentinel-2 (**C**) data. The central panels refer to the Sentinel-1 (**D**) ALOS-2 (**E**) and Sentinel-2 (**F**) post-seismic images whereas the bottom ones show the products of data processing technique applied on Sentinel-1 (**G**), ALOS-2 (**H**) and Sentinel-2 (**I**) data. The red polygon represents the affected area (see Figure 10).

**Figure 9:**
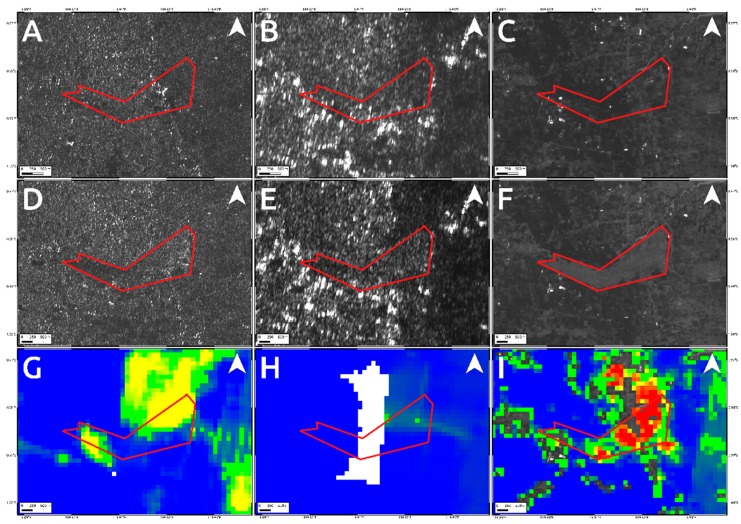
Data comparison for constraining the landslides activated by the M_w_ 7.5 2018 Sulawesi earthquake in the eastern outskirts of the Palu city. Upper panels show the pre-seismic image for Sentinel-1 (**A**), ALOS-2 (**B**) and Sentinel-2 (**C**) data. The central panels refer to the Sentinel-1 (**D**), ALOS-2 (**E**) and Sentinel-2 (**F**) post-seismic images whereas the bottom ones show the products of data processing technique applied on Sentinel-1 (**G**), ALOS-2 (**H**) and Sentinel-2 (**I**) data. The red polygon represents the affected area (see Figure 10).

**Figure 10:**
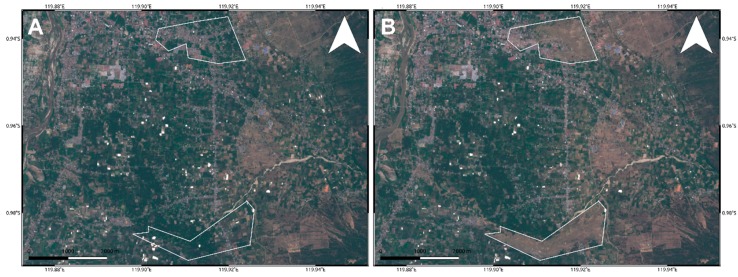
Landslides captured by Sentinel-2 data in RGB color representation. Pre- (**A**) and post-seismic (**B**) image.

**Figure 11:**
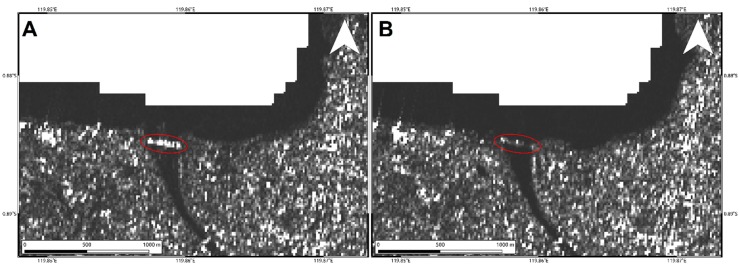
Sentinel-1 pre- (**A**) and post-seismic (**B**) intensity SAR images showing the Palu IV bridge collapse.

**Figure 12:**
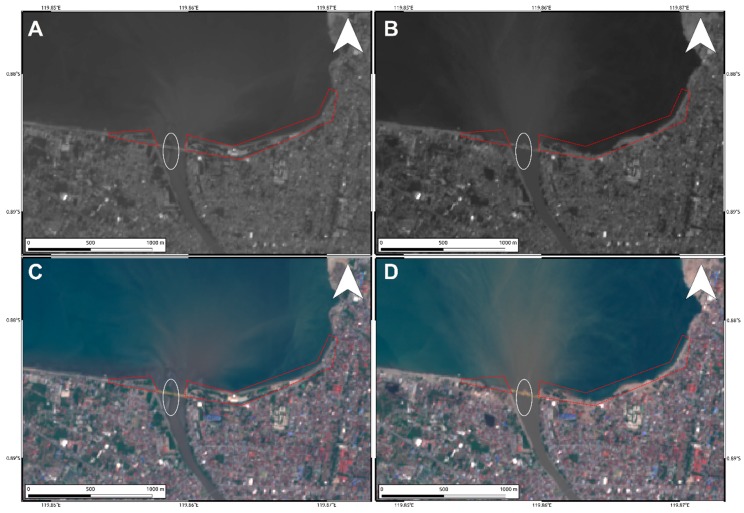
Pre- (**A**,**C**) and post-seismic (**B**,**D**) Sentinel-2 optical images. The coastline retreat is clearly visible in red polygons. The white ellipse indicates the small island at the river mouth completely disappeared in the post-seismic images.

To analyze such secondary effects, in addition to the results shown in Figure 4 and Figure 5, we also exploited the pre- and post-event intensity SAR and optical images since this parameter is sensitive to any changes in the investigated scenario. Concerning the two landslides, optical data work much better than SAR ones, showing the induced phenomena both considering a visual analysis and the Sub-Pixel correlation (SBP) processing products where local deformations surrounded by the mainshock signal are observed. Conversely, SAR intensity data are too much affected by speckle noise and POT outcomes seem to be unreliable to detect such events due to the coarse resolution (Figure 8, Figure 9 and Figure 10). On the contrary, along Palu bay, the information provided by SAR backscattering signal between pre-and post-seismic images produces different brightness values that allow to well recognize the Palu IV bridge collapse (Figure 11). On the other hand, the coastline retreat is better constrained by observing the comparison between pre- and post-seismic optical data, especially considering the Red Green Blue (RGB) color representation (Figure 12). 

## 5. Discussion

Both SAR and optical remote sensing data processed by POT and SBP techniques well constrain the strong displacement field due to the mainshock of the M_w_ 7.5 Sulawesi earthquake. The deformation pattern retrieved from different data is the same and is consistent with the left-lateral NNW–SSE geometry of the causative Palu-Koro fault. The retrieved results are also in agreement with the ones shown in some previous works. Indeed, in [32], Sentinel-2 and Planet images were processed by different software [25,33] to estimate a co-seismic offset of 5.4m in the proximity of Palu urban area. Similar outcomes with Sentinel-2 images were obtained by Soquet et al. [12] using Co-registration of Optically Sensed Images and Correlation (COSI-Corr) software packages [34] and also by Bao et al. [11] with another image correlation method [35]. In the latter, POT analysis with ALOS-2 Fine Beam data was also performed showing a maximum slip of about 6m near the city of Palu and a difference in the magnitude of average slip between the northern unmapped segment (~1.9 m) and the known southern segment (~4.7 m) of the Palu-Koro fault. In all cases, remote sensing data revealed how the seismic event produced the most significant co-seismic slip along the sector of the Palu-Koro Fault just south the urban area of Palu whereas the northern unknown segment slipped with smaller values.

One of the main difference between SAR and optical products is related to the nature of the operating sensor. Indeed, optical sensors are passive sensors since they acquire the sun radiation backscattered from the ground thus not operating during the night. On the other hand, SAR sensors are day and night operating, being active and sun-light independent sensors.

**Figure 13:**
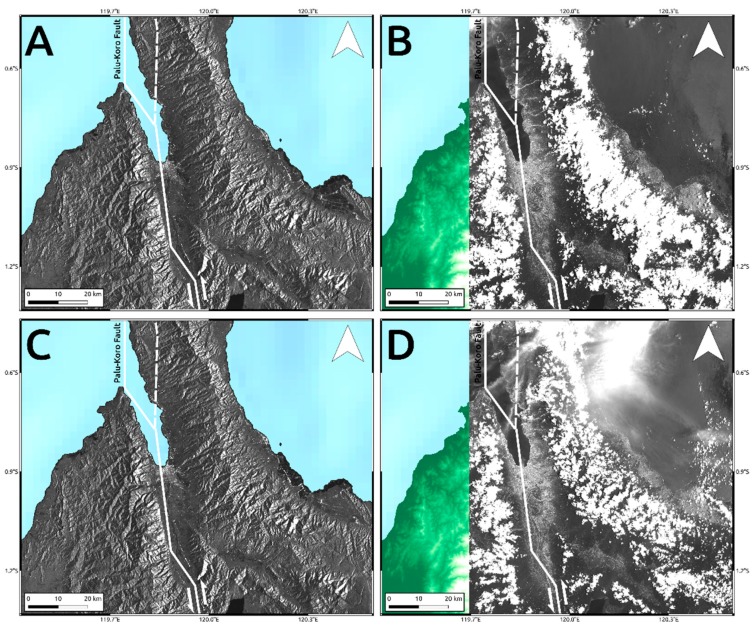
Comparison between Sentinel-1 SAR (**A**,**C**) data and Sentinel-2 optical intensity images (**B**,**D**). Pre- (**A**,**B**) and post-event (**C**,**D**) images. Solid lines are the main tectonic structures crossing the island retrieved by the GEM Global Active Faults project [16]. The dashed line is the unmapped segment of the Palu-Koro fault that ruptured during the seismic event.

In addition, because of the smaller wavelength (λ~390–700 nm), the presence of clouds is a significant source of error affecting optical data whereas SAR sensors are not sensitive to such phenomena and work in any weather conditions (λ~3–30 cm). Considering the investigated scenario, most of signal loss observed in optical results of Figure 5 is just because of the cloud coverage (Figure 13B) which is instead completely invisible at SAR microwaves (Figure 13A). 

Conversely, because of the speckle effect, SAR intensity images are noisier than the optical ones, being formed by coherent interaction of the transmitted microwave signal with the ground targets. Since there are several scatterers distributed randomly in each pixel, at this resolution, it leads to a random noise affecting both the visual inspection on intensity images and the detection by POT technique of small scale phenomena such as the two landslides induced in the surrounding of Palu urban area (Figure 8 and Figure 9). A strategy to reduce SAR speckle noise is to average the images by multi-looking operation. However, the choice of multi-look factors is not straightforward and sometimes such operation is not suitable for improving the visual inspection on intensity images since big factors lead to greater pixel spacing, i.e., coarser resolutions, that prevent the observation of local-scale deformation phenomena. At the same time, in POT-based analysis, the presence of such random noise severely impact on the cross-correlation estimation, especially considering windows matching size consistent with the spatial dimension of relatively small phenomena, since common features between images could be not found. Moreover, random bright targets could dominate the cross-correlation function thus generating false matches. Therefore, in order to increase the probability to find common features for estimating the cross-correlation function in SAR images, we used larger matching window size than optical data (1500 m vs. 70 m). Obviously, in such way, we can only observe the displacement due to the mainshock masking the induced landslides phenomena. In particular, it is clearly shown in L-band ALOS-2 SAR data where the main signal due to the Palu-Koro fault slip is predominant. Sentinel-1 SAR data show some deformation gradients in the proximity of the two landslides but as stated before and also shown in Figure 4A, there are several noising signals surrounding the mainshock therefore, it is not possible to be confident about them. Instead, such phenomena are better constrained by optical data. SBP Sentinel-2 products show, in Figure 5 and Figure 8I and Figure 9I, very localized signals whose geometry and entity make them most likely ascribable to the two big landslides. In addition, a visual inspection on the full resolution (~10 m) image further validate such hypothesis (Figure 8C,F; Figure 9C,F; and Figure 10).

Regarding the damages along the coastal area of Palu bay, SAR intensity seems to be more appropriate for identifying the Palu IV bridge collapse. This is due to the different backscattering values of SAR signal reflected by the bridge and the surrounding water. Before the collapse, the bridge is represented by a “stripe” of very bright targets clearly distinguishable with respect to the low intensity of the surrounded water (Figure 11A). Obviously, after the collapse of the bridge in the water (see Figure 7) such different behavior is no longer defined (Figure 11B). Optical data are not so sensitive to such phenomenon although they show very well the consequences of the seismic-induced events such as tsunamis, soil liquefaction, and coastal landslides, i.e., a significant coastal retreat and the disappearance of the small island below the bridge (Figure 12). They are also shown in SAR data but are less emphasized because of speckle noise and the coarser resolution with respect to optical data (~15 m vs. ~10 m) (Figure 11). 

## 6. Conclusions

We exploited remote sensing SAR and optical data to quantitatively evaluate the Palu-Koro fault slip caused by the 2018 M_w_ 7.5 Sulawesi earthquake and analyze some seismic-induced phenomena. Standard Differential SAR interferometry technique has demonstrated to be not suitable in the study of the NS striking mechanism of the earthquake. Consequently, we adopted the POT and SBP methods that despite their not very high resolution and high accuracy, are the most suitable approaches for inferring ground deformation field in such particular motion directions. 

Although the different data sources, the results in terms of NS co-seismic displacement field are in very good agreement and are both consistent with in-situ analysis [26]. Optical data are normally characterized by a better spatial resolution thus preserving more details especially considering small scale phenomena. In addition, they can provide more accurate products when used to map displacements by means of cross-correlation methods as the one proposed in this work. However, they are sunlight dependent and are also strongly affected by the cloud coverage. Conversely, SAR sensors work in any atmospheric conditions during both night and day being active sensors. Moreover, based on some SAR backscattering signal properties, several phenomena can be detected by evaluating the behavior of parameters such as amplitude, phase, coherence and so on. However, SAR speckle noise strongly affects the measurements preventing the small scale phenomena evaluation. For these reasons, the synergic and complementary use of SAR and optical data is strongly encouraged to improve the study of such kind of phenomena. Recently, such strategy is also suggested by the Copernicus program of the ESA [36] that includes in their objectives the exploitation of twin missions, i.e., Sentinel-1 and Sentinel-2, able to provide data with similar spatial coverage, resolution and revisit time. 

## Figures and Tables

**Table 1:** Zonal statistics of the deformation pattern estimated by Sentinel-1, ALOS-2 (SAR), and Sentinel-2 (optical) sensors.

Satellite	Mean [m]	Min [m]	Max [m]	*σ* [m]
ALOS-2	0.40	−4.73	5.88	1.62
Sentinel-1	0.43	−4.15	4.70	1.90
Sentinel-2	0.31	−4.8	6.5	1.49
